# Multiomics characterization of breast angiosarcoma from an Asian cohort reveals enrichment for angiogenesis signaling pathway and tumor-infiltrating macrophages

**DOI:** 10.3389/fimmu.2024.1515935

**Published:** 2025-01-13

**Authors:** Tun Kiat Ko, Zexi Guo, Bavani Kannan, Boon Yee Lim, Jing Yi Lee, Zhimei Li, Elizabeth Chun Yong Lee, Bin Tean Teh, Jason Yongsheng Chan

**Affiliations:** ^1^ Cancer Discovery Hub, National Cancer Centre Singapore, Singapore, Singapore; ^2^ Laboratory of Cancer Epigenome, National Cancer Centre Singapore, Singapore, Singapore; ^3^ Oncology Academic Clinical Program, Duke-NUS Medical School, Singapore, Singapore; ^4^ Division of Medical Oncology, National Cancer Centre Singapore, Singapore, Singapore

**Keywords:** sarcoma, next generation sequencing, immunotherapy, tumor-infiltrating macrophages, immune-oncology

## Abstract

**Introduction:**

Recent epidemiological data suggests a rising incidence of breast angiosarcoma (AS-B) in the Western population, with over two-thirds related to irradiation or chronic lymphedema. However, unlike head and neck angiosarcoma (AS-HN), AS-B disease characteristics in Asia remain unclear.

**Methods:**

We examined clinical patterns of angiosarcoma patients (n = 176) seen in an Asiantertiary cancer center from 1999 to 2021, and specifically investigated the molecular and immune features of AS-B in comparison to AS-HN. Data from whole genome sequencing (WGS), NanoString gene expression profiling and 10x Genomics Visium spatial transcriptomics were analyzed.

**Results:**

Majority of cases were AS-HN (n = 104; 59.1%), while AS-B (n = 16, all females) accounted for 9.1% of the cases. The median age at diagnosis was 43 years (range, 26 to 74). Based on WGS, 4 of the 7 AS-B had non-synonymous somatic variants in 47 genes (range, 2 to 28 per case). These genes were functionally annotated and were enriched in cancer-related pathways such as regulation of cell differentiation, VEGFR and receptor tyrosine kinases signaling pathways. By NanoString gene expression profiling, ASB, compared to AS-HN, were enriched for angiogenesis, notch signaling and metastasis-associated matrix remodeling pathways. Additionally, AS-B were enriched for macrophages and CD8+ T cells expression signatures. Similarly, Visium spatial transcriptomics showed that AS-B were enriched for macrophages and T-cells.

**Discussion:**

In conclusion, in our AS-B cases, we observed a convergence of both mutational and expression signatures on angiogenic-related pathways. Thus, anti-angiogenic therapy could be an option to treat AS-B.

## Introduction

Angiosarcoma is a highly aggressive endothelial-cell tumor that accounts for 1-2% of soft tissue sarcomas and 5.4% of cutaneous sarcomas in humans (1). Despite its rarity, population-based data indicate a rising incidence over recent decades, particularly among older adults. Prognosis of angiosarcoma is dismal largely due to its tendency for local recurrence and distant metastasis, with a reported 5-year overall survival rate of 30-50% (2, 3). The management and treatment of angiosarcoma is a significant clinical challenge. While surgery remains as the primary curative method, achieving wide surgical margins is problematic due to diffuse tissue infiltration and anatomical constraints particularly in regions such as the head and neck. With post-surgery local recurrence rates ranging from 35-86%, adjuvant radiotherapy is often administered; however, the effectiveness of this approach remains uncertain (4, 5). Despite high risks of metastasis in angiosarcoma, there are limited studies on the effectiveness of chemotherapy as well.

The most common sites for angiosarcoma are the cutaneous areas, particularly in the head and neck regions (6), and this subtype has been extensively studied (7–9). Nonetheless, angiosarcoma can also arise in any region of the body. The increasing use of breast-conserving surgery and radiation therapy in breast cancer management has contributed to an increased incidence of secondary breast angiosarcoma (AS-B). This is particularly evident in cases accompanied by the onset of chronic lymphedema (Stewart-Treves syndrome). Secondary AS-B comprises 0.09% to 0.16% of breast cancer diagnoses and often emerge in older women, with the peak occurrence observed in their 60s, while primary AS-B accounts for approximately 0.04% of breast cancer cases and typically affects women in their 30s to 40s. Both primary and secondary AS-B tend to be more aggressive than mammary carcinoma, with a greater likelihood of both local recurrence and distant metastasis. Generally, the prognosis for AS-B is generally less favorable compared to more common types of breast cancer (10, 11).

Angiosarcoma presents as a heterogeneous disease, exhibiting distinct genomic and immune microenvironment profiles across various anatomical subtypes (12–14). Due to the rarity of AS-B, information on the genetic and transcriptomic landscapes of the disease is limited, with only two reported cases of transcriptomic analyses in primary AS-B (15, 16). A study by Espejo-Freire et al. indicated that head and neck angiosarcoma (AS-HN) exhibited a higher prevalence of *TP53* (50%) and *POT1* (40.5%) mutations compared to other subtypes, while AS-B were more likely to harbor *MYC* amplification (63.3%). AS-HN also demonstrated immunotherapy response biomarkers such as high tumor mutational burden and PD-L1 positivity (14). This highlights the potential for targeted and personalized therapeutic strategies through subtype-specific profiling of angiosarcoma. In this study, we conducted a retrospective analysis of Asian angiosarcoma patients, focusing on AS-HN (n = 104) and AS-B (n = 16), examining subtype-specific clinical and molecular aspects. We aim to investigate the distinctive molecular profiles of AS-B and AS-HN, including mutational and expression signatures, immuno-oncologic pathways, and characteristics of the tumor microenvironment.

## Materials and methods

### Patient cohort

Patients diagnosed with histologically-proven angiosarcoma (n = 176) at the Singapore General Hospital (SGH) and National Cancer Centre Singapore (NCCS) between 1999 and 2021 were included in our study. All of the cases were reviewed by certified pathologists and the diagnoses were supported by immunohistochemical staining for vascular markers such as CD31 and/or ERG. Kaposi sarcoma, epithelioid hemangioendothelioma, and intimal sarcoma were excluded from the study. In this study, we focused on angiosarcoma of the breast (AS-B, n = 16) and angiosarcoma of the head and neck region (AS-HN, n = 104). Age, sex, and ethnicity of the patients were corroborated against their National Registry Identification Cards. All data were obtained at the time of diagnosis or subsequent follow-up. Clinico-pathological characteristics of all patients with breast and head and neck angiosarcoma are summarized in **Table 1**.

**Table 1:** Clinical characteristics of angiosarcoma patients in the study cohort.

	AS-HN n (%)	AS-B n (%)	*p*
Total	104 (100%)	16 (100%)	
Age (years)			
• Mean ± SD • Median (range)	73.7 ± 12.6 74.4 (27.8 - 104)	44.9 ± 16.2 42.6 (26.4 – 74.1)	< 0.0001
Sex			
• Male • Female	74 (71.2%) 30 (28.8%)	0 (0%) 16 (100%)	< 0.0001
Ethnicity			
• Chinese • Malay • Indian • Others	91 (90.4%) 6 (5.8%) 2 (1.9%) 5 (4.8%)	8 (50.0%) 3 (18.8%) 1 (6.3%) 4 (25.0%)	0.0028
Known secondary cause			
• Yes • No	1 (0.8%) 103 (99.2%)	4 (25.0%) 12 (75.0%)	0.00102
Stage at diagnosis (%)			
• Metastatic • Non-metastatic	33 (33.0%) 67 (67.0%)	0 (0%) 16 (100%)	0.00535

AS-HN, angiosarcoma of the head and neck; AS-B, angiosarcoma of the breast.

Missing data: Stage at diagnosis: AS-HN, n=4.

### Whole genome sequencing (WGS) and somatic variant calling

Whole genome sequencing (WGS) were analyzed for AS-B (n =7), using our previously published dataset (9). Briefly, samples were sequenced either on the Illumina HiSeq X platform as paired-end 150-base pair reads using DNA inserts averaging 350 bp (NovogeneAIT Genomics Singapore Pte Ltd), or using MGI DNBSEQ technology (DNA nanoball sequencing platform by MGI Tech, China) based on 100 bp paired-end reads, on the MGI DNBSEQ-G400 platform (MGI Tech, China). Variant calling was performed as previously described (9).

### NanoString gene expression profiling

Gene expression profiling on FFPE tissue was performed using the NanoString PanCancer IO360 panel on the nCounter platform (NanoString Technologies, Seattle, WA, USA) following manufacturer’s protocol. RNA was extracted from five 10 μm sections on all samples with adequate tumor tissue available and analyzed using the 2100 Bioanalyzer (Agilent Technologies, Palo Alto, CA, USA). The final cohort (AS-HN, n = 42; AS-B, n = 7) was analyzed on the nSolver 4.0 Advanced Analysis module using default settings to derive differentially expressed genes, pathway scores, and cell-type scores. Briefly, gene expression was normalized for top 10 best normalization probes. For differential expression of genes, the optimal model was selected and p-values were adjusted by the method of Benjamini-Yekutieli. For cell type profiles, a p-value threshold of 0.05 was adopted for reporting a cell type’s results. The panel was then analyzed using the TIS (Tumour Inflammation Signature) algorithm, which measures inflammation and immune activation within the tumor environment (17). The signature includes 18 genes related to adaptive immune resistance, cytotoxic activity, chemokine expression, and antigen presentation. A TIS score is calculated through a weighted linear combination of the 18 genes’ expression values, with each gene’s expression value normalized to a stable housekeeper gene. Samples are considered to exhibit a more inflamed or “hot” tumor phenotype if their TIS scores are greater than or equal to the median score of the cohort.

### 10x Genomics Visium CytAssist platform

Formalin-fixed, paraffin-embedded (FFPE) tissue blocks of two AS breast samples, one primary (AS-05) and one secondary (AS-42) were chosen based on DV200% scores for the Visium CytAssist Spatial Gene Expression for FFPE assay (10X Genomics, USA). Briefly, 10 μm sections were collected for RNA extraction and QC with the RNeasy FFPE Kit (Qiagen, Germany) and the High Sensitivity RNA ScreenTape assay (Agilent Technologies, USA), respectively. Samples with the two highest DV200% were chosen and further assessed for histology QC with hematoxylin and eosin (H&E) staining. The chosen blocks were then sectioned at 5 μm thickness onto plain, positively charged slides (Leica Biosystems, Germany). Deparaffinization, H&E staining and imaging, followed by decrosslinking were performed as per manufacturer’s protocol (CG000520). For the gene expression assay, probes targeting the human whole transcriptome were added to the tissue, followed by ligation of the hybridized probe pairs. The Visium CytAssist instrument was then used to transfer the released probes from the plain charged slides onto the Visium CytAssist Spatial Gene Expression slide with 11 x 11 mm capture areas. Probe extension, elution and subsequent library preparation steps are performed on bench top according to the rest of the Visium CytAssist Spatial Gene Expression Reagent Kits User Guide (CG000495). Final libraries were profiled using the Agilent Bioanalyzer High Sensitivity DNA kit (Agilent Technologies, USA). Loupe Browser 6.0 was used to estimate the percentage of capture area covered by the tissue within each frame on the slide to calculate the sequencing depths required. The libraries were sent for paired-end dual-indexed sequencing for a minimum of 25,000 read pairs per spot on the Novaseq 6000 instrument (Illumina).

### Analysis of spatial sequencing data

Spatial transcriptomic profiling was performed on the 10x Genomics Visium platform as previously described (18). The reads were demultiplexed and mapped against the hg38 reference genome using 10X Space Ranger v.1.3.1 (10x Genomics, CA, USA) with the default parameters for automatic alignment. Using Seurat v4.0, spatial data were first loaded into count matrices, and spots which had less than 10% of transcripts mapping to mitochondrial genes were retained for the scaling and normalization of genes expression measurements using sctransform approach. Selected genes (tumor cells: PECAM1 and ERG; fibroblasts: FBLN1, FAP, and DES; B-cells: CD79A and CD79B; CD8 T-cells: CD8A and CD8B; NK-cells: KLRK1 and KLRD1; macrophages: CD14 and CD68; neutrophils: CSF3R and FPR1, Dendritic cells: CD209 and CCL13 and Mast cells: TPSAB1, MS4A2, CPA3, and HDC) were used to annotate immune cells.

### Statistics

Comparisons of the frequencies of categorical variables were performed using Pearson’s Chi-squared tests or Fisher’s exact tests. Continuous variables were compared with student’s t-test. Overall survival (OS) was calculated from the date of diagnosis to the date of death from any cause, or was censored at the date of last follow-up for survivors. Relapse-free survival (RFS) was defined as the time elapsed from the date of diagnosis till the date of relapse or death from any cause. Survival analyses were conducted using the Kaplan Meier method and log-rank test. All statistical analyses were conducted assuming a two-sided test with a significance level of 0.05 unless otherwise stated and were performed using MedCalc for Windows version 19.0.4 (MedCalc Software, Ostend, Belgium).

### Study approval

Written informed consent for the use of biospecimens and clinical data was obtained in accordance with the Declaration of Helsinki. The study was approved by the SingHealth Centralised Institutional Review Board (CIRB 2010/426/B).

## Results

### Clinical characteristics and patient outcomes

**Figure 1:**
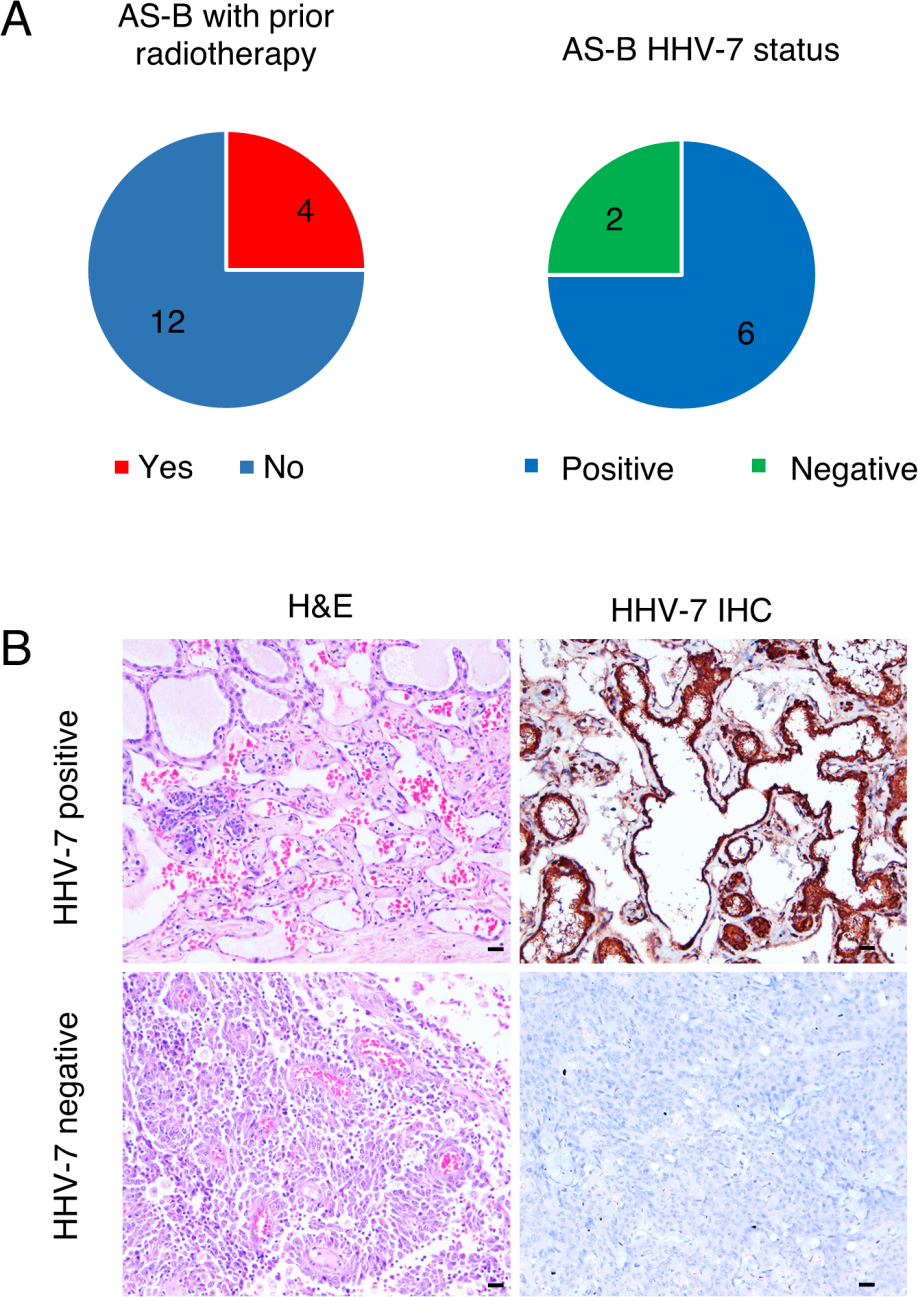
Overview of AS-B cases in the study. Pie charts showing: **(A)** the number of AS-B patients in our cohort with prior radiotherapy (left) and the number of evaluated AS-B patients that were immunostained positive for HHV7 (right). **(B)** Representative images of AS-B samples that were either stained with H&E or immunostained with HHV7-specific antibody.

**Figure 2:**
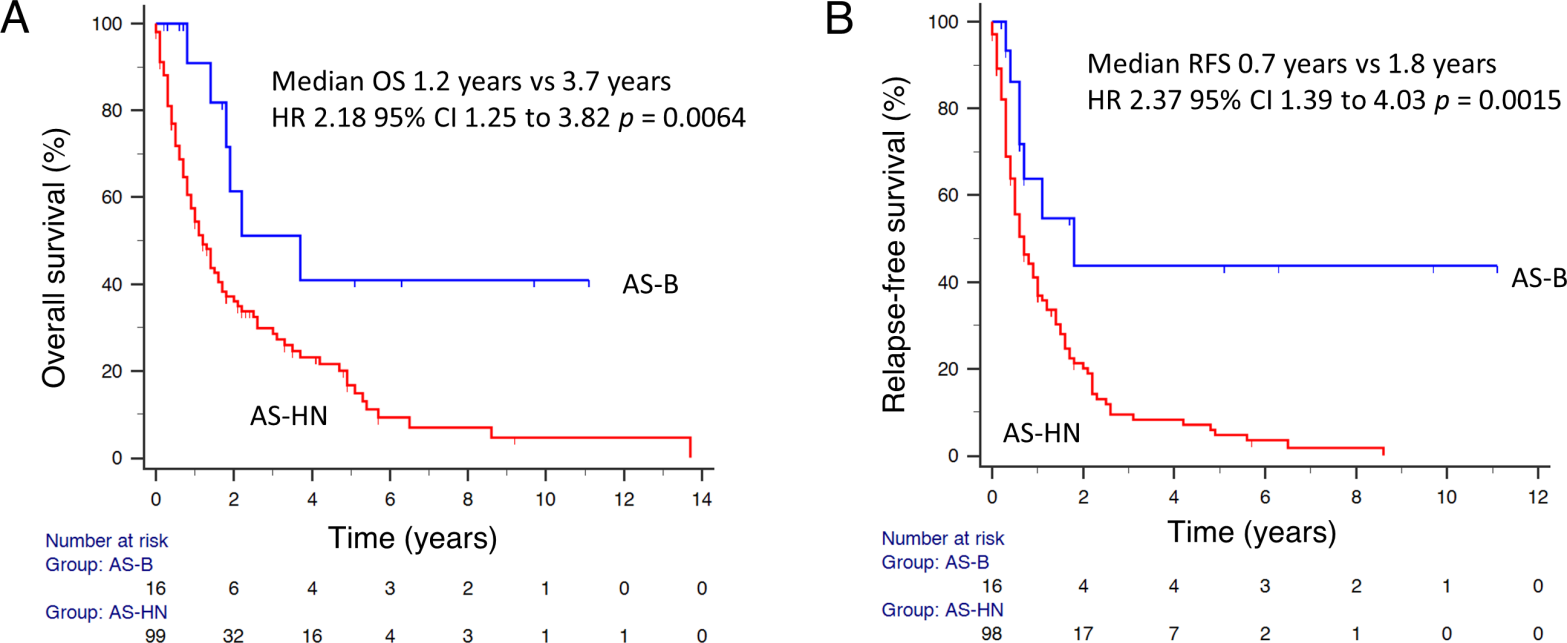
Survival outcomes of patients with AS-B. **(A)** Overall survival and **(B)** relapse-free survival data were shown for AS-HN and AS-B in our cohorts.

A total of 176 patients were included in our study. Most cases (n = 104, 59.1%) were of primary head and neck origin (AS-HN), while AS-B (n = 16) accounted for 9.1% of the cases (**Table 1**). The median age of patients with AS-B (42.6 years; range, 26.4 – 74.1) was significantly lower than those with AS-HN (74.4 years; range, 27.8 – 104) (*p* < 0.0001). AS-HN comprised mostly men (71.2%) while all AS-B occurred in women (*p* < 0.0001). Notably, while patients with AS-HN were mostly of Chinese ethnicity (90.4%), only 50% of patients with AS-B were Chinese, followed by Malay (18.8%), Indian (6.3%) and others (25%) (*p* = 0.0028). Among AS-B, four cases (25%) were related to prior irradiation, while 6 out of 8 cases (75%) evaluated were positive for human herpesvirus-7 (HHV-7) as determined previously on immunohistochemistry (**Figure 1**) (12). Although all cases were non-metastatic at diagnosis and were treated with curative-intent surgery, 6 patients (37.5%) experienced rapid relapse within a median of 6.0 months. Median RFS for AS-HN was 0.7 years compared to 1.8 years for AS-B (HR 2.37, 95% CI 1.39 to 4.03, *p* = 0.0015), while median OS was 1.2 years and 3.7 years, respectively (HR 2.18, 95% CI 1.25 to 3.82, *p* = 0.0064) (**Figure 2**).

### Somatic mutational landscape of breast angiosarcoma

**Figure 3:**
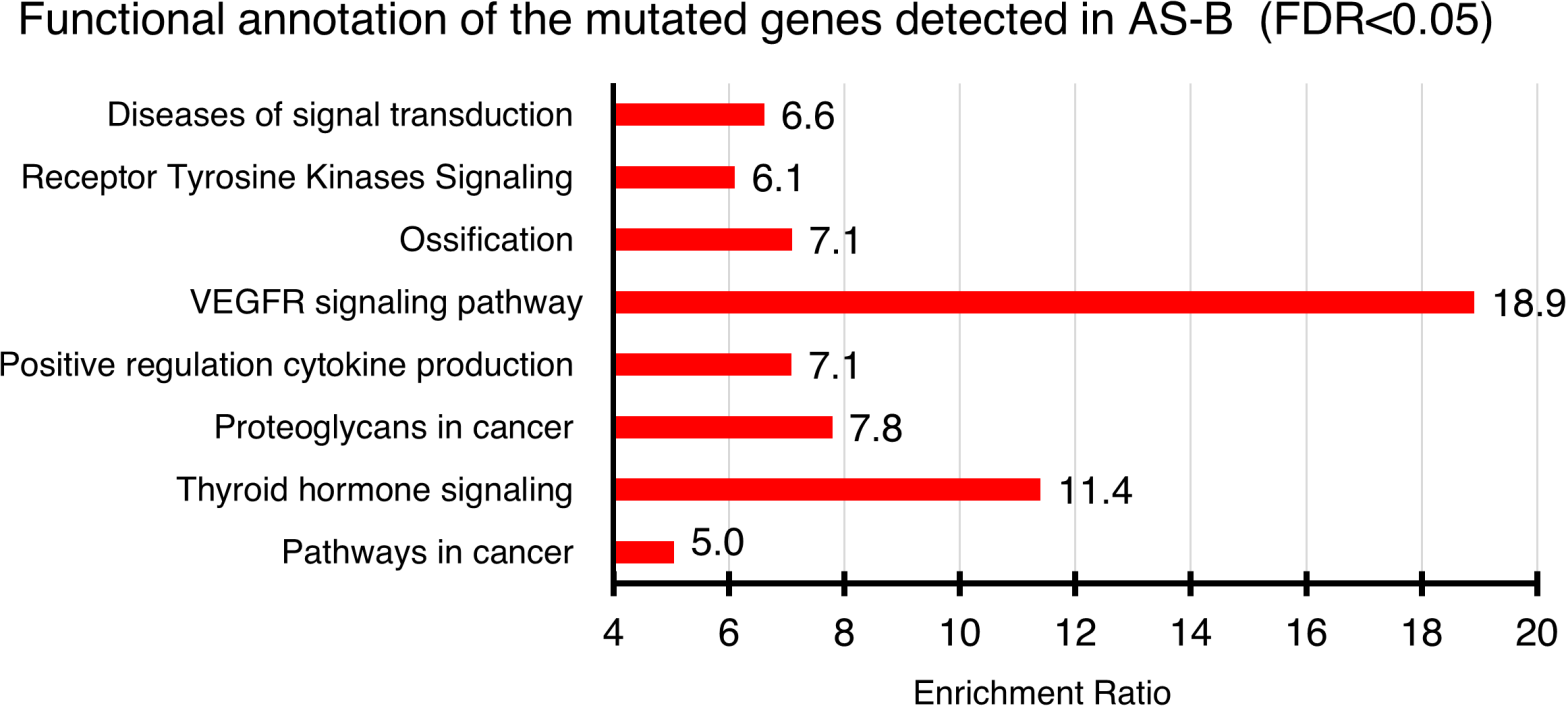
Functional annotation of genes found mutated in AS-B samples. Annotation were done by using WEBGESTALT (WEB-based GEne SeT AnaLysis Toolkit) which interrogates publicly available databases (e.g. Gene Ontology, KEGG & Reactome). Only statistically significant (FDR < 0.05) pathways were shown. The enrichment ratio for each of the enriched pathways were shown.

Based on WGS data (**Supplementary Table S1**), 4 of the 7 (57.1%) AS-B samples evaluated had non-synonymous somatic variants in a total of 47 genes (range, 2 to 28 per case). Among the notable variants found were in kinases (*KDR*, *JAK2*, *FLT4*, *ATR*, *EPHB1* & *PRKACA*). Other variants were found in genes, when impaired, are known to cause cancers (e.g. *ATRX, FOXO1, SMAD3, PTCH1, NOTCH4, HIF1A, SETD1A, CREB1* and *GAB1)*. KDR was the only gene with recurrent hits (n = 2). Based on our recently published on AS-HN, we found 373 non-synonymous variants (9). We found that there were 27 genes that were common to both our AS-B and AS-HN samples (**Supplementary Table S2**). These included KDR, FLT4, NOTCH4, ATRX & HIF1A. These 47 genes were functionally annotated by using WebGestalt (WEB-based GEne SeT AnaLysis Toolkits) over representation analysis (ORA) (https://www.webgestalt.org) on publicly available databases (e.g. Gene Ontology and KEGG). These 47 genes were found to be enriched in VEGFR signaling pathway as well as other cancer-related pathways such as regulation of cell differentiation, cytokine regulation and receptor tyrosine kinases signaling pathways (**Figure 3**).

Additionally, by performing WebGestalt-based ORA, we found that these genes were also significantly enriched in highly cited cancer gene databases such as COSMIC Cancer Gene Census tier 1 (FDR= 1.44E-11, Enrichment= 3.48) and OncoKB (FDR= 1.18E-11, Enrichment= 2.26) (**Supplementary Tables S3**, **S4**). We had also curated, from selected published datasets, variants that had been reported in AS-B cases (**Supplementary Table S3**). Some of these published datasets were based on targeted panels (**Supplementary Table S3**). While others were based on whole genome sequencing (WGS) and whole exome sequencing (WES) (**Supplementary Table S3**). Based on WebGestalt-based ORA, our AS-B variants were significantly enriched in four of these published AS-B datasets (**Supplementary Table S4**; FDR range: 2.07E-5 to 0.031, Enrichment range: 4.76 to 10.53).

### Immune and oncogenic signaling pathways in breast angiosarcoma

**Figure 4:**
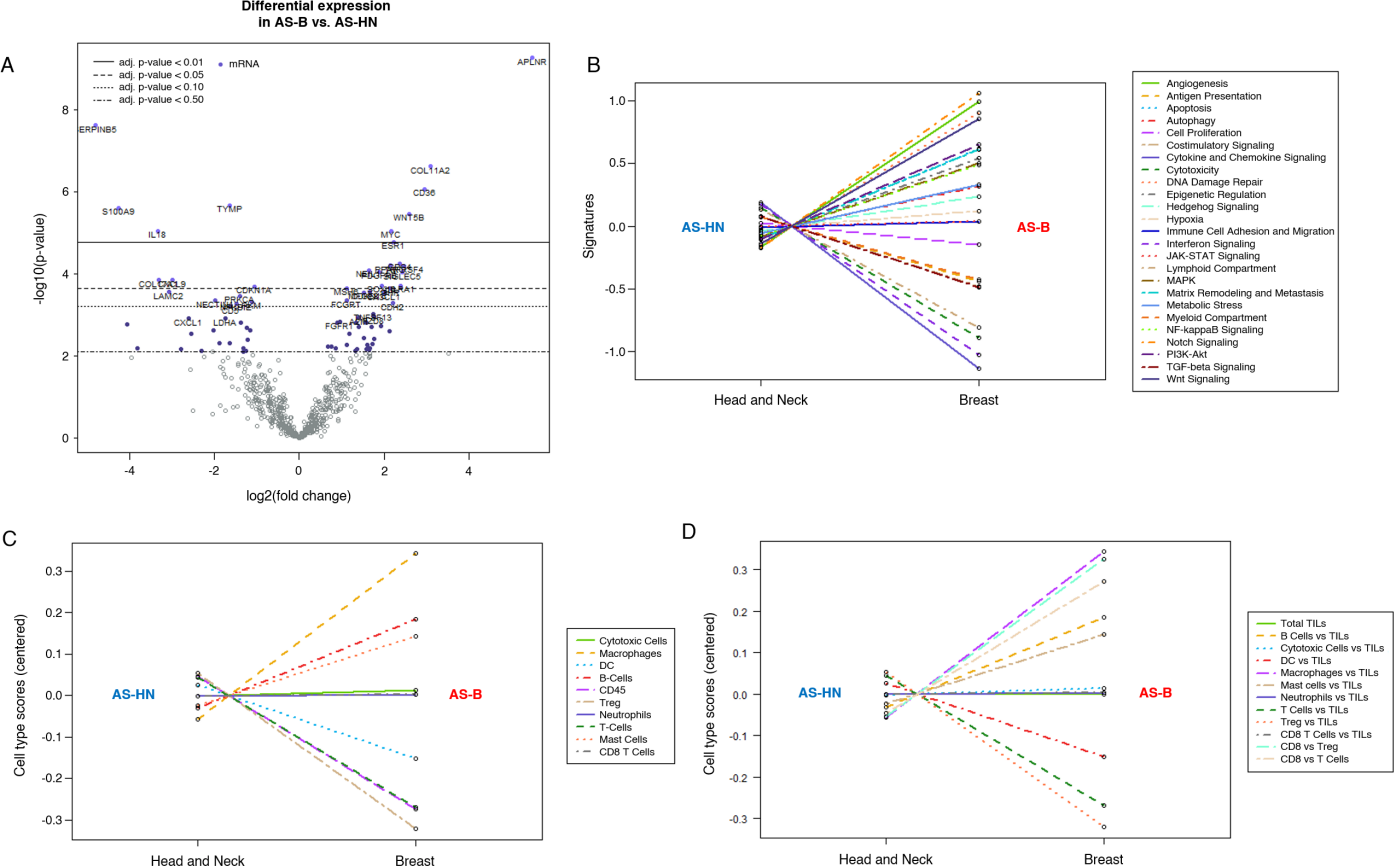
Nanostring IO360 RNA expression profiling comparing AS-B samples to AS-HN samples. **(A)** Volcano plot showing genes that were statistically significant and differentially expressed. Line graphs indicating signaling pathways **(B)** and immune cells, raw **(C)** and relative **(D)** covariates, that were either enriched or depleted in AS-B when compared to AS-HN.

NanoString PanCancer IO360 770-gene expression profiling (AS-HN, n = 42; AS-B, n = 7) platform was used to determine the oncogenic and immune pathways that were enriched in AS-B tumor samples when compared to those from AS-HN. Among the transcripts that were significantly upregulated (log2 fold change > 1, FDR < 0.05) include well-known oncogenes such as the pro-angiogenic apelin receptor (*APLNR*), *WNT5B*, *TNFRSF4*, *MYC*, *PDGFRB* and *ESR1*. Transcripts that were significantly downregulated (log2 fold change < -1, FDR < 0.05) in AS-B include tumor suppressors such as *CDKN1A* and anti-angiogenic *SERPINB5*; cytokines such as *IL18*, *CXCL9* as well as *S100A9* which is an immune and inflammation regulator (**Figure 4A**; **Supplementary Table S5**). The top 3 pathways that were enriched in AS-B were Notch signaling, angiogenesis and DNA damage repair. Conversely, the top 3 pathways that were depleted in AS-B were cytokine and chemokine signaling, interferon signaling and cytotoxicity (**Figure 4B**). We also performed WebGestalt-based geneset enrichment analysis (GSEA) on the 664 differentially expressed genes between AS-B and AS-HN (**Supplementary Table S5**). We interrogated 3 different publicly available databases namely the TCGA sarcoma (TCGA_SARC), TCGA invasive breast carcinoma (TCGA_BRCA) and the mSigDb (Molecular Signatures Database) Hallmark50 (**Supplementary Tables S6**-**S8**). Based on these databases, we found that AS-B were significantly enriched (FDR<0.05) for angiogenic related genesets (**Supplementary Tables S6**-**S8**). Conversely, AS-B were depleted of immune-related genesets such as immune effector, interferon gamma and interferon alpha (**Supplementary Tables S6**-**S8**). These findings was in agreement with the Nanostring-based findings (**Figure 4A**). Next, we bioinformatically profiled for the presence of tumor–infiltrating immune cells in AS-B and AS-HN samples by using the Nanostring-based immune cell type-specific gene expression signatures. Compared to AS-HN, AS-B samples were found to be enriched for macrophage gene expression signature. Additionally, AS-B samples were enriched for gene expression signature of CD8+ T cell but were depleted for gene expression signature of T-reg cell (**Figures 4C, D**). TIS scores ranged from 6.06 to 7.57 (median, 6.81).

### Spatial analysis of breast angiosarcoma

**Figure 5:**
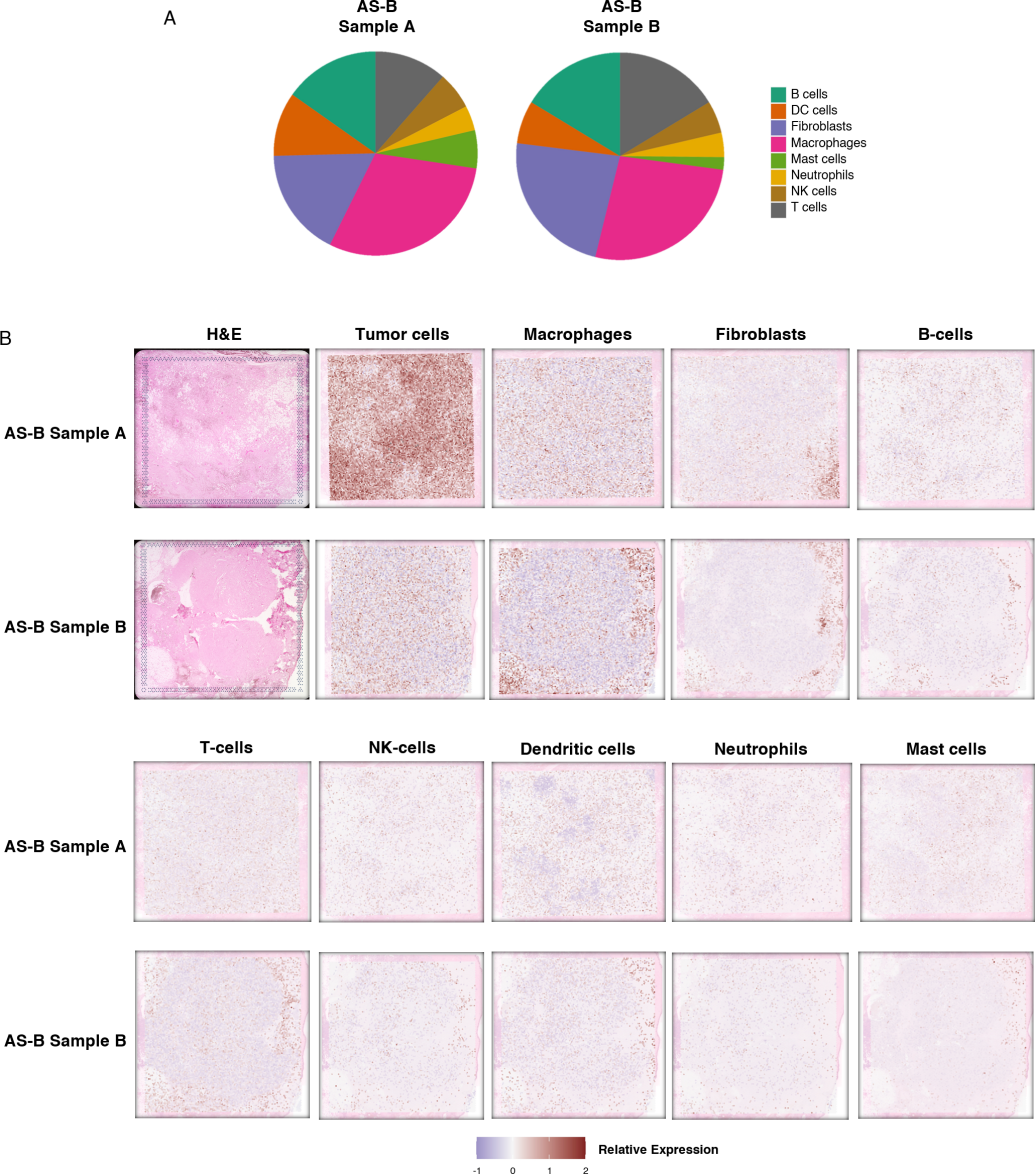
Spatial transcriptomics data showed that macrophages are the most prevalent immune cells in the AS-B tumor microenvironment. **(A)** Venn diagrams showing the proportions of the different types of immune cells detected. **(B)** Figure panels showing H&E images, along with images illustrating the estimated distribution of the tumor cells, fibroblasts and various types of immune cells detected in each sample.

Subsequently, we used 10x Genomics Visium spatial transcriptomics to analyze two AS-B samples. Spatial transcriptomics analysis showed that sample A had more tumor cells when compared to sample B. The most prevalent immune cells in both AS-B samples were macrophages. Other non-tumor cells observed, in order of abundance, include fibroblasts, B cells, dendritic cells and T-cells. In sample A, capture sites that were enriched for macrophages were found among those that were enriched for tumor cells. In sample B, most of the sites that were enriched macrophages were in the periphery of the tumor. The same trend was observed for B-cells, dendritic cells and T-cells (**Figure 5**). We had recently published spatial transcriptomics data, using the 10x Genomics Visium platform, on 4 AS-HN samples (9). In these AS-HN samples, macrophages were among the most abundant immune cells. Similar to AS-B sample A, macrophages were located among the tumor cells in 3 of the AS-HN samples (9). Similarly, in these 3 AS-HN samples, other immune cells such as T cells, B cells and dendrtic cells were found among the tumor cells, albeit at different level of abundance (9).

## Discussion

Based on WGS performed on our AS-B cohort, we have observed that KDR is the most recurrent hit. Other published genomic data had shown that KDR was a recurrent hit among their AS-B samples as well as in other type of AS (9, 12, 19–21) (**Supplementary Tables S3**, **S4**). We found that by functionally annotating all the variants detected in our AS-B cohort, these variants were enriched for angiogenesis as well as other cancer-related pathways. Additionally, we found that gene expression profiling, by Nanostring, of our AS-B cohort showed that angiogenesis was among the top enriched pathway. We subsequently used GSEA to interrogate publicly available gene expression datasets (e.g. TCGA RNA-seq data and mSigDB Hallmark 50) and also found that AS-B samples were enriched for angiogenic-related pathways but were depleted of immune-related pathways. These findings were similar to those from the Nanostring platform. Furthermore, it had been reported that angiogenic pathways was enriched in other AS transcriptome cohorts (12, 22). Thus, we observed a convergence of both mutational & expression signatures on the angiogenic signaling pathway. Therefore, the mutational and gene expression results showed that VEGFR inhibitors and other angiogenic pathways inhibitors might be useful candidates for treating AS-B patients.

We have also analyzed the immune landscape of AS-B by using the NanoString immune cell profiling by gene expression signature which indicated that the main immune cells in AS-B were macrophages. Furthermore, based on NanoString gene expression signatures, CD8+ T cells are the predominant tumor-infiltrating lymphocytes or TILs in AS-B. Conversely, we found that AS-B samples were depleted of the gene expression signature of the immunosuppressive and tumor-promoting Treg cells. Our 10x Genomics Visium spatial transcriptomic data also corroborated with our Nanostring gene expression data and revealed that AS-B tumors were enriched for tumor-infiltrating macrophages. These tumor-infiltrating macrophages might turn out to be tumor-associated macrophages or TAMs. Even though they could have tumor promoting properties, these macrophages could potentially be immunomodulated to become tumor killing provided the right therapy is used (23). Our current immune landscape data on AS-B had provided valuable insights on the location of various immune cell type within the AS-B tumors. In order to validate and to expand on our current findings on the immune landscape, our future endeavors would have to include the use of next generation spatial transcriptomic platforms such as 10x Genomics VisiumHD and BGI STOMICS.

## Data Availability

The NanoString gene expression profiling data and spatial transcriptomic data used in the current study are available in GEO under accession no. GSE226338 and GSE227469.

## References

[1] Lim RMH Lee JY Kannan B Ko TK Chan JY. Molecular and immune pathobiology of human angiosarcoma. Biochim Biophys Acta (BBA) Rev Cancer. (2024) 1879:189159. doi: 10.1016/j.bbcan.2024.189159 39032539

[2] Rouhani P Fletcher CDM Devesa SS Toro JR. Cutaneous soft tissue sarcoma incidence patterns in the U.S. Cancer. (2008) 113:616–27. doi: 10.1002/cncr.v113:3 18618615

[3] Buehler D Rice SR Moody JS Rush P Hafez GR Attia S. Angiosarcoma outcomes and prognostic factors: A 25-year single institution experience. Am J Clin Oncol. (2014) 37:473. doi: 10.1097/COC.0b013e31827e4e7b 23428947 PMC3664266

[4] Young RJ Brown NJ Reed MW Hughes D Woll PJ. Angiosarcoma. Lancet Oncol. (2010) 11:983–91. doi: 10.1016/S1470-2045(10)70023-1 20537949

[5] Chan JY Tan GF Yeong J Ong CW Ng DY Lee E. Clinical implications of systemic and local immune responses in human angiosarcoma. NPJ Precis Onc. (2021) 5:1–13. doi: 10.1038/s41698-021-00150-x PMC7881182 33580206

[6] Patel SH Hayden RE Hinni ML Wong WW Foote RL Milani S. Angiosarcoma of the scalp and face: the mayo clinic experience. JAMA Otolaryngology–Head Neck Surg. (2015) 141:335–40. doi: 10.1001/jamaoto.2014.3584 25634014

[7] Ramakrishnan N Mokhtari R Charville GW Bui N Ganjoo K. Cutaneous angiosarcoma of the head and neck—A retrospective analysis of 47 patients. Cancers. (2022) 14:3841. doi: 10.3390/cancers14153841 35954504 PMC9367417

[8] Guan L Palmeri M Groisberg R. Cutaneous angiosarcoma: A review of current evidence for treatment with checkpoint inhibitors. Front Med (Lausanne). (2023) 10:1090168. doi: 10.3389/fmed.2023.1090168 36993810 PMC10040781

[9] Loh JW Lee JY Lim AH Guan P Lim BY Kannan B. Spatial transcriptomics reveal topological immune landscapes of Asian head and neck angiosarcoma. Commun Biol. (2023) 6:1–9. doi: 10.1038/s42003-023-04856-5 37106027 PMC10140281

[10] Zemanova M Machalekova K Sandorova M Boljesikova E Skultetyova M Svec J. Clinical management of secondary angiosarcoma after breast conservation therapy. Rep Pract Oncol Radiother. (2013) 19:37–46. doi: 10.1016/j.rpor.2013.07.013 24936318 PMC4056516

[11] Esposito E Avino F di Giacomo R Donzelli I Marone U Melucci MT. Angiosarcoma of the breast, the unknown—a review of the current literature. Transl Cancer Res. (2019) 8:S510–7. doi: 10.21037/tcr.2019.07.38 PMC8798946 35117129

[12] Chan JY Lim JQ Yeong J Ravi V Guan P Boot A. Multiomic analysis and immunoprofiling reveal distinct subtypes of human angiosarcoma. J Clin Invest. (2020) 130:5833–46. doi: 10.1172/JCI139080 PMC7598061 33016928

[13] van Ravensteijn SG Versleijen-Jonkers YM Hillebrandt-Roeffen MH Weidema ME Nederkoorn MJ Bol KF. Immunological and genomic analysis reveals clinically relevant distinctions between angiosarcoma subgroups. Cancers. (2022) 14:5938. doi: 10.3390/cancers14235938 36497420 PMC9739001

[14] Espejo-Freire AP Elliott A Rosenberg A Costa PA Barreto-Coelho P Jonczak E. Genomic landscape of angiosarcoma: A targeted and immunotherapy biomarker analysis. Cancers. (2021) 13:4816. doi: 10.3390/cancers13194816 34638300 PMC8507700

[15] Rincón-Riveros A De la Peña J Rubiano W Olivella F Martinez-Agüero M Villegas VE. Primary breast angiosarcoma: comparative transcriptome analysis. Int J Mol Sci. (2022) 23:16032. doi: 10.3390/ijms232416032 36555675 PMC9781631

[16] Matsunuma R Sato S Chan JY Kinoshita K Taku K Yamaguchi K. Enrichment of immune-related genes in aggressive primary breast angiosarcoma: A case report. Case Rep Oncol. (2023) 16:461–70. doi: 10.1159/000531490 PMC10368090 37497424

[17] Danaher P Warren S Lu R Samayoa J Sullivan A Pekker I. Pan-cancer adaptive immune resistance as defined by the Tumor Inflammation Signature (TIS): results from The Cancer Genome Atlas (TCGA). J Immunother Cancer. (2018) 6:63. doi: 10.1186/s40425-018-0367-1 29929551 PMC6013904

[18] Tai SB Lee EC Lim BY Kannan B Lee JY Guo Z. Tumor-infiltrating mast cells in angiosarcoma correlate with immuno-oncology pathways and adverse clinical outcomes. Lab Invest. (2024) 104:100323. doi: 10.1016/j.labinv.2024.100323 38218317

[19] Painter CA Jain E Tomson BN Dunphy M Stoddard RE Thomas BS. The Angiosarcoma Project: enabling genomic and clinical discoveries in a rare cancer through patient-partnered research. Nat Med. (2020) 26:181–7. doi: 10.1038/s41591-019-0749-z 32042194

[20] Huang S-C Zhang L Sung YS Chen CL Kao YC Agaram NP. Recurrent CIC Gene Abnormalities in Angiosarcomas: A Molecular Study of 120 Cases With Concurrent Investigation of: PLCG1:: KDR:: MYC: and: FLT4: Gene Alterations. Am J Surg Pathol. (2016) 40:645. doi: 10.1097/PAS.0000000000000582 26735859 PMC4833528

[21] Murali R Chandramohan R Möller I Scholz SL Berger M Huberman K. Targeted massively parallel sequencing of angiosarcomas reveals frequent activation of the mitogen activated protein kinase pathway. Oncotarget. (2015) 6:36041–52. doi: 10.18632/oncotarget.v6i34 PMC4742160 26440310

[22] Rosenbaum E Antonescu CR Smith S Bradic M Kashani D Richards AL. Clinical, genomic, and transcriptomic correlates of response to immune checkpoint blockade-based therapy in a cohort of patients with angiosarcoma treated at a single center. J Immunother Cancer. (2022) 10:e004149. doi: 10.1136/jitc-2021-004149 35365586 PMC8977792

[23] Mantovani A Allavena P Marchesi F Garlanda C. Macrophages as tools and targets in cancer therapy. Nat Rev Drug Discovery. (2022) 21:799–820. doi: 10.1038/s41573-022-00520-5 35974096 PMC9380983

